# Uncovering the Mechanism of Astragalus membranaceus in the Treatment of Diabetic Nephropathy Based on Network Pharmacology

**DOI:** 10.1155/2020/5947304

**Published:** 2020-03-02

**Authors:** Ming-Fei Guo, Ya-Ji Dai, Jia-Rong Gao, Pei-Jie Chen

**Affiliations:** ^1^Department of Pharmacy, The Fourth Affiliated Hospital of Anhui Medical University, Hefei, Anhui 230012, China; ^2^Department of Pharmacy, Anhui No.2 Provincial People's Hospital, Hefei, Anhui 230041, China; ^3^Department of Pharmacy, The First Affiliated Hospital of Anhui University of Chinese Medicine, Hefei, Anhui 230031, China

## Abstract

**Background:**

Diabetic nephropathy (DN), characterized by hyperglycemia, hypertension, proteinuria, and edema, is a unique microvascular complication of diabetes. Traditional Chinese medicine (TCM) *Astragalus membranaceus* (AM) has been widely used for DN in China while the pharmacological mechanisms are still unclear. This work is aimed at undertaking a network pharmacology analysis to reveal the mechanism of the effects of AM in DN. *Materials and Methods*. In this study, chemical constituents of AM were obtained via Traditional Chinese Medicine Systems Pharmacology Database (TCMSP), and the potential targets of AM were identified using the Therapeutic Target Database (TTD). DisGeNET and GeneCards databases were used to collect DN-related target genes. DN-AM common target protein interaction network was established by using the STRING database. Gene Ontology (GO) and Kyoto Encyclopedia of Genes and Genomes (KEGG) pathway enrichment analyses were carried out to further explore the DN mechanism and therapeutic effect of AM. The network diagrams of the active component-action target and protein-protein interaction (PPI) networks were constructed using Cytoscape software.

**Results:**

A total of 16 active ingredients contained and 78 putative identified target genes were screened from AM, of which 42 overlapped with the targets of DN and were considered potential therapeutic targets. The analysis of the network results showed that the AM activity of component quercetin, formononetin, calycosin, 7-O-methylisomucronulatol, and quercetin have a good binding activity with top ten screened targets, such as VEGFA, TNF, IL-6, MAPK, CCL3, NOS3, PTGS2, IL-1*β*, JUN, and EGFR. GO and KEGG analyses revealed that these targets were associated with inflammatory response, angiogenesis, oxidative stress reaction, rheumatoid arthritis, and other biological process.

**Conclusions:**

This study demonstrated the multicomponent, multitarget, and multichannel characteristics of AM, which provided a novel approach for further research of the mechanism of AM in the treatment of DN.

## 1. Introduction

The etiology and pathogenesis of diabetic nephropathy (DN) are complex and diverse [1]. There are many factors involved, such as infection, autoimmunity, genetics, drugs, diet, and environment [2, 3]. The specific pathogenesis is still not fully clear. The incidence of DN accounts for 20%-40% of all diabetic patients, and it is also one of the main causes of death [4]. DN is characterized by edema, hyperglycemia, hypertension, and albuminuria. With the deterioration of DN, it will eventually lead to glomerulosclerosis and renal failure. Therefore, lowering the blood sugar, decreasing blood pressure, and alleviation of renal microvascular damage are crucial for the treatment of DN.

Traditional Chinese medicine (TCM) has been playing an important role in the improvement of life quality and curing disease. In recent years, much attention has been focused on the Chinese herb medicine for the treatment of diabetes and metabolic syndrome. *Astragalus membranaceus* (AM) has a longstanding history and has gained widespread clinical applications in China, with the functions of lowering blood sugar, decreasing blood lipid, eliminating edema, antioxidative stress, and so on [5, 6]. In addition, it has been proven to have wide pharmacological effects on diabetic diseases; particularly in DN aspects, it has a unique curative effect [7, 8].

It is well known that TCM is guided by the theory of TCM and characteristics of being multicomponent, multitarget, and multipathway in the treatment of diseases. AM therapeutic effects on DN by regulating Nrf2/HO-1 signaling pathway, antioxidant stress, nuclear factor-kappa B, and immunoregulation were reported [9, 10]. However, the pharmacological mechanisms and material bases of the action of AM related to DN remain elusive. Accordingly, an increasing number of researchers are keen to explore comprehensive and systematic evaluation of the pharmacological mechanism of AM on DN which is urgently needed.

**Figure 1:**
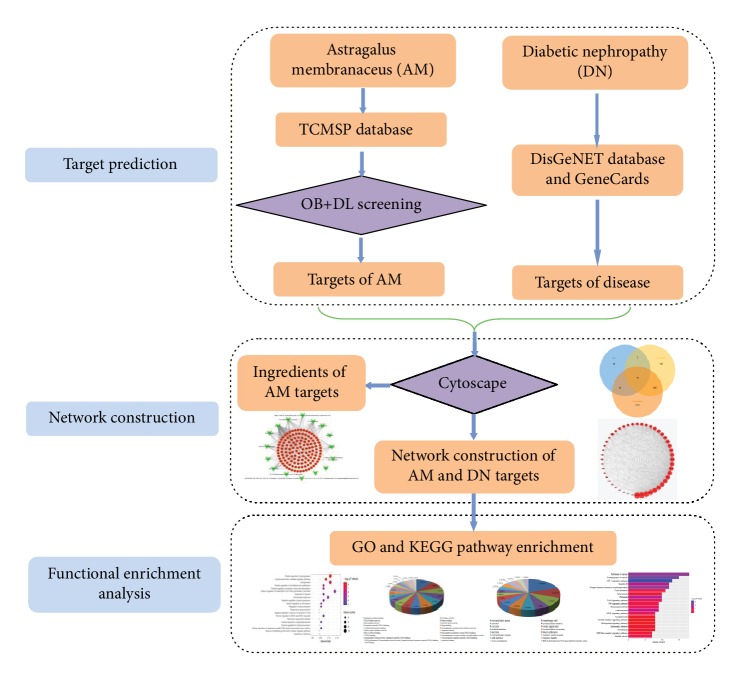
The whole framework based on an integration strategy of network pharmacology.

Network pharmacology is an interactive network based on the drug-target-gene-disease, including chemoinformatics, bioinformatics, network biology, and pharmacology [11, 12]. It has become a comprehensive tool to systematically reveal the complex network relationships between the bioactive components and potential mechanisms of TCM formulas from a systemic perspective [13]. The purpose of this study was to explore the potential mechanism of AM on DN disease by using network pharmacology, drug-targeted interaction databases, and biological analysis methods. Our flowchart is shown in Figure 1.

## 2. Materials and Methods

### 2.1. Database Building and Active Compound Screening

Traditional Chinese Medicine System Pharmacology Database (TCMSP, http://lsp.nwu.edu.cn/tcmsp.php) was used to collect the chemical constituents of AM; it was a unique system pharmacology platform that captures the relationships between herbal ingredients, targets, and diseases [14, 15]. It is constructed based on scientific publications and medical literature on TCM, which contains more than 13,731 pure compounds isolated from 505 TCM herbs. Twelve pharmacokinetic parameters can be provided, such as oral bioavailability (OB), half-life (HL), drug-likeness (DL), Caco-2 permeability (Caco-2), and blood-brain barrier (BBB). We screened AM compounds based on absorption, distribution, metabolism, and excretion (ADME), and pharmacokinetic information retrieval filters were used to retrieve bioactive compounds for further analysis under the conditions of OB ≥ 30% and DL ≥ 0.18 [16, 17].

### 2.2. Screening of Potential Targets for DN

Information on DN-associated target genes were gathered from the following database. DisGeNET (https://www.disgenet.org/home/), a comprehensive multifunctional data platform that contains integrated diseases, genes, and experimental research [18]. The GeneCards (https://www.genecards.org/) as a comprehensive database of functions including genomics, proteomics, and transcriptomics [19]. Therefore, we searched in these two databases with the keywords “Diabetic nephropathy” to screen the targets related to DN. The names of targets and their ID were searched on Therapeutic Target Database (TTD) (http://db.idrblab.org/ttd/). It provides information regarding the known and explored therapeutic protein targets, the targeted disease, and the corresponding ID at each of the targets.

### 2.3. Collecting Compound-Disease Common Targets

The screened chemical targets and disease targets database were imported into the ImageGP (http://www.ehbio.com/ImageGP/index.php) platform for analysis, and the common targets of compound-disease were obtained as the potential targets for further analysis [20].

### 2.4. PPI Network Map of Compound-Disease Common Targets

Protein-protein interaction (PPI) network was derived based on STRING database (https://string-db.org/), which covered almost all functional interactions between the expressed proteins [21]. Species are set as “Homo sapiens” and the target interaction information was obtained according to the results of analysis. The results were imported into the Cytoscape (version 3.6.1; https://www.cytoscape.org/) software where the interaction network was drawn and analyzed. The node size was used to reflect the number of combined targets (degree), and the degree greater than twice the median degree of all nodes was selected as a hub according to our previous studies.

### 2.5. Construction of Active Component-Target Network

A visual network was established through Cytoscape software to reflect the complex relationship between active compounds and potential targets [22]. Nodes represent the compounds and targets, while edges indicate the intermolecular interactions between compounds and targets.

### 2.6. Gene Ontology (GO) and KEGG Pathway Enrichment Analysis

The biological process (BP), molecular function (MF), cell component (CC), and Kyoto Encyclopedia of Genes and Genomes (KEGG) database (https://www.kegg.jp/) pathway enrichment analysis were using the DAVID system (https://david.ncifcrf.gov/) [23, 24]. In this research, GO functional annotation and KEGG pathway enrichment analyses were performed using R package, and the *P* value less than 0.05 was employed for further analysis.

## 3. Results

### 3.1. Active Compounds of AM

The active compound targets of AM were searched via the TCMSP databases for each chemical component. Eighty-seven compounds were collected from the TCMSP, with the values of OB ≥ 30% and DL ≥ 0.18 properties, applied to screen the active compounds from AM. At last, 16 candidate ingredients were selected out from AM compounds (Table 1). In total, 142 targets were identified after removing the duplicate data (Supplementary [Supplementary-material supplementary-material-1]).

### 3.2. Compound-Target Network

**Figure 2:**
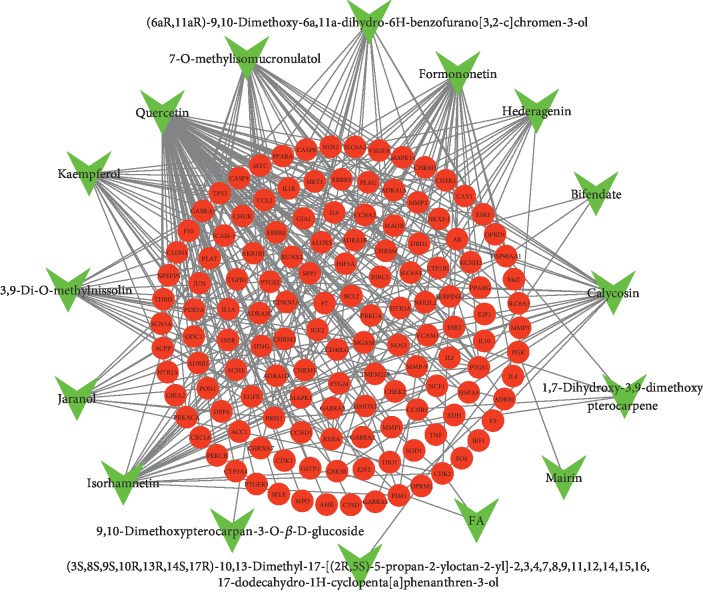
Compound-target network of potential targets in AM. The light green nodes represent the potential active ingredients in AM, and the red nodes represent the corresponding targets of the ingredients.

To further understand the interaction relationship between the compounds and their corresponding targets, we constructed a compound-target network, as shown in Figure 2. By mapping 16 compounds to 142 potential targets which associated with antioxidant stress, nuclear factor-kappa B, and immune regulation. Based on the docking results, the network embodied 151 nodes and 296 edges, in which the colorized red circles correspond to the targets and the compounds calculated in green from AM. The compounds with more targets are quercetin, 7-O-methylisomucronulatol, formononetin, and isorhamnetin, which correspond to 99, 32, 32, and 23 targets, respectively. The result suggested that these four components probably served as significant therapeutic compounds in DN.

### 3.3. Retrieval of Potential Disease Targets

**Figure 3:**
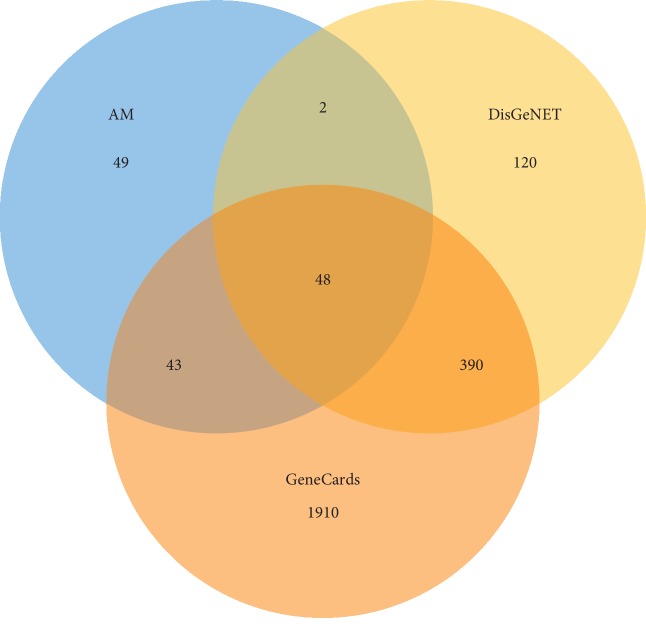
Matching of target genes between DN and AM.

Through the DisGeNET and GeneCards databases, the retrieved results were integrated to obtain the DN-related disease protein targets. As shown in Figure 3, the potential target gene in AM was mapped to the disease target gene using the ImageGP platform, and a Venn diagram was drawn. A total of 48 potential targets were obtained based on the intersection of protein targets acting on chemical components and these are related to DN (Table 2). The information regarding these targets is provided in Supplementary [Supplementary-material supplementary-material-1].

### 3.4. Conversion of Target Proteins into Network and Analysis

**Figure 4:**
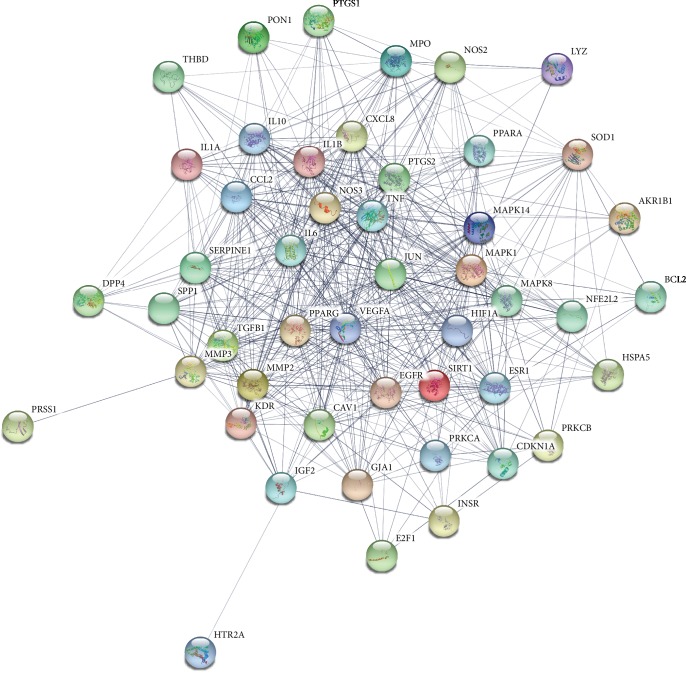
Common target PPI network between DN and AM. Each bubble node represents a protein, and the 3D structure in the bubble nodes represent that the protein spatial structure is known or predicted. The lines among inner nodes display the relationship between different proteins, and the width of lines was based on the strength of data support.

**Figure 5:**
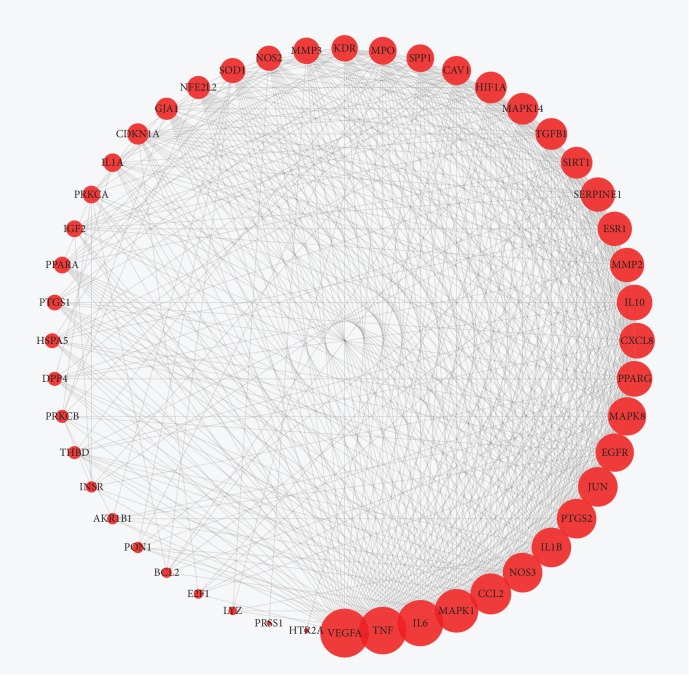
The PPI network of targets for AM in the treatment of DN. The layout of the outer ring is according to the area and color of nodes, which goes better in an anticlockwise direction. The red nodes represent the potential target of AM in DN. The size of the nodes is shown in a gradient from large to small according to the descending order of the degree value. And, the lines among inner nodes display the relationship between different proteins.

A total of 48 potential genes associated with DN were uploaded to the STRING database for analysis. The systematically selected protein targets with a medium confidence score of 0.400 were plotted as an interaction network. The network of protein-protein interactions (PPI) (Figure 4) was established through the STRING database. From the analysis results, a total of 48 nodes and 539 edges were acquired, and the average node degree is 22.5. The results were used for further analysis through Cytoscape software, and the network was constructed as Figure 5. The edges represent the association between a pair of action targets, the nodes represent the action target, and the degree value represents its action intensity. The top ten targets VEGFA, TNF, IL-6, MAPK1, CCL2, PTGS2, IL-1*β*, NOS3, JUN, and EGFR have higher degree in this process, which explained their significance in the network.

### 3.5. Gene Ontology Enrichment Analysis

**Figure 6:**
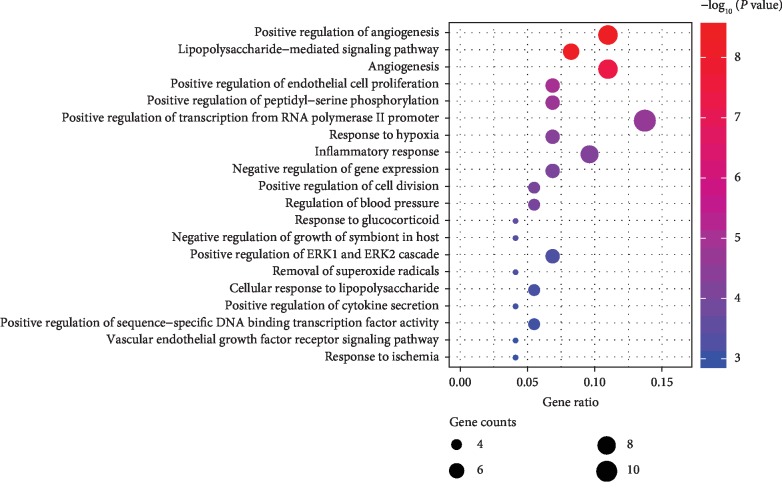
Enriched GO terms for biological process (BP) of potential targets of the main active ingredients of AM. The color of the nodes is shown in a gradient from red to blue according to the descending order of the *P* value. The size of nodes is arranged according to the ascending order of the number of gene counts.

We imported the selected potential 48 target genes into the DAVID system for GO analysis. The results revealed that the functions of these potential targets are related to many biological processes, molecular function, and cellular components. It may be important for the occurrence and development of DN. A total of 139 biological processes were enrichment, and the top 20 remarkably enriched BP terms were selected for analysis, such as positive regulation of angiogenesis, inflammatory response, and removal of superoxide radicals (Figure 6). The results indicated that AM may play a vital role in the treatment of DN by manipulating these biological processes. These processes are of great significance to further understand the mechanism of AM on the DN.

**Figure 7:**
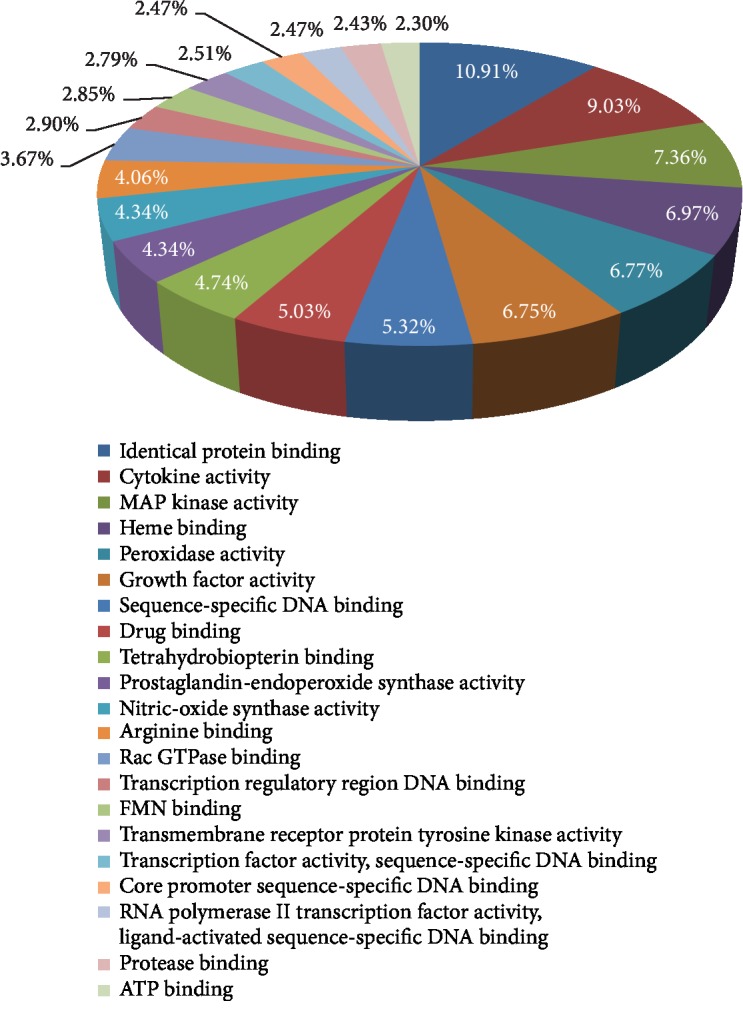
Enriched GO terms for molecular function (MF) of potential targets of the main active ingredients of AM. The percentage of MF is clockwise arranged according to the descending order of the *P* value.

A total of 21 molecular function (Figure 7) GO terms were enrichment, and the top 13 entries were selected based on *P* < 0.05. These targets of molecular function mainly involved identical protein binding, cytokine activity, MAP kinase activity, heme binding, peroxidase activity, growth factor activity, and many genes related to the molecular functions described above.

**Figure 8:**
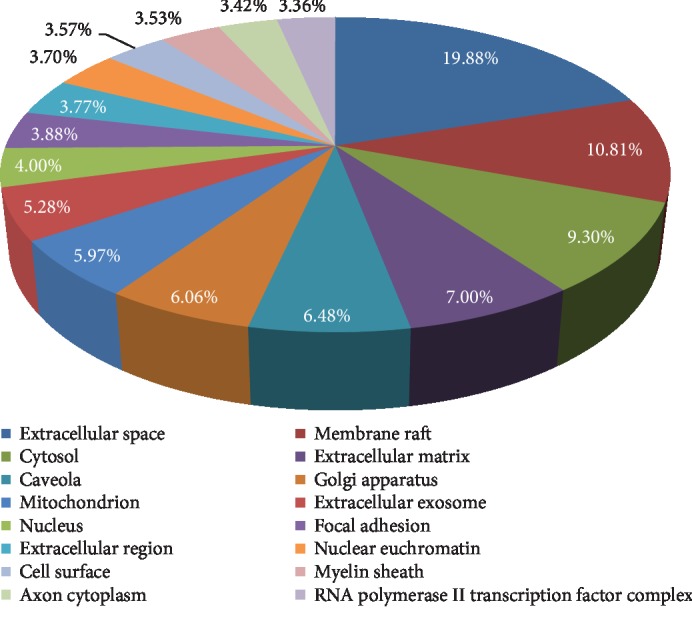
Enriched GO terms for cell component (CC) of potential targets of the main active ingredients of AM. The percentage of CC is clockwise arranged according to the descending order of the *P* value.

In all, 16 cell component (Figure 8) GO terms were enrichment, and the top 12 entries were selected based on *P* < 0.05. The targets are closely related in extracellular space, membrane raft, cytosol, extracellular matrix, caveola, Golgi apparatus, mitochondrion, and extracellular exosome, and many targets were ranked highly as potential related genes.

### 3.6. KEGG Pathway Enrichment Analysis

**Figure 9:**
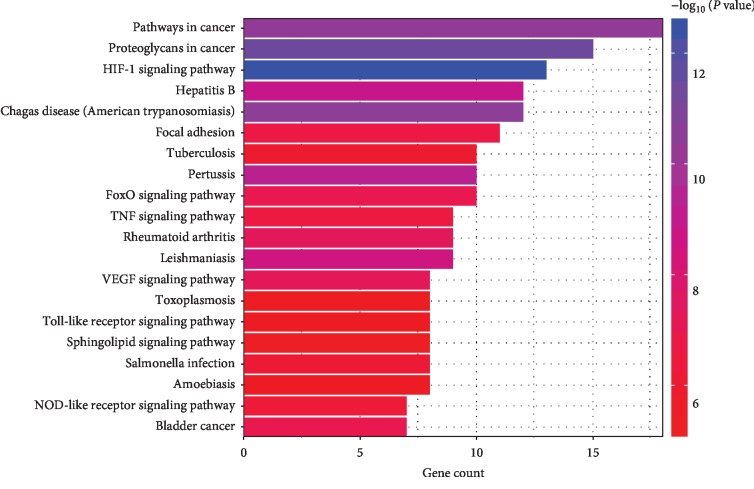
KEGG pathway analysis of putative target genes of AM. The color of the item is shown in a gradient from blue to red according to the descending order of the *P* value. The length of item is arranged according to the ascending order of the number of gene counts.

To further reveal the potential mechanism of the AM on the effect of DN, we conducted KEGG pathway enrichment analysis on 48 targets and screened out top 20 pathways based on the threshold of *P* < 0.01 (Figure 9). Numerous pathways for potential target genes were identified, such as VEGF signaling pathway, HIF-1 signaling pathway, and FoxO signaling pathways which are associated with signal transduction. Rheumatoid arthritis and TNF signaling pathways are related to the inflammatory reaction process. Bladder cancer and proteoglycans play a crucial role in the pathways in cancer. Moreover, toll-like receptor signaling pathway and focal adhesion are closely related to immunological stress. In addition, we found some other pathways such as leishmaniasis, salmonella infection, amoebiasis, and tuberculosis, which revealed that AM has a potential application in other related diseases. The results certificated that AM alleviated the DN disease by regulating antioxidant stress, immunization, and inflammatory reaction.

## 4. Discussion

Diabetic nephropathy (DN) is one of the common chronic microvascular complications in diabetes mellitus. At present, the incidence of DN is increasing year by year, which seriously threatens the prognosis and quality of life in diabetic patients [25]. The occurrence and development of DN are related to the disorder of glucose and lipid metabolism, oxidative stress, inflammatory reaction, and abnormal vasoactive substances [26, 27]. In this study, the TCM-active ingredient-target network showed that the main active ingredient of AM quercetin, 7-O-methylisomucronulatol, formononetin, and isorhamnetin plays an important role in the network, indicating that they have potential research value in the treatment of DN.

Evidence showed that some compounds found in this study not only have anti-immune and antistress effects but also have the effect of alleviating endocrine and metabolic disorders. It was reported that quercetin could inhibit glomerular mesangial cell proliferation in high glucose-treated DN mouse and via reactivation of the Hippo pathway [28]. Meanwhile, quercetin can increase the SIRT1 expression and resistance to oxidative stress [29]. Previous studies have suggested that formononetin could significantly decrease the HIF-1 and VEGF protein expression level, which is widely used in the field of cardiovascular diseases [30, 31].

As we can see from the compound-target network, many targets can be adjusted by multiple compounds, such as NOS3, PTGS2, JUN, MAPK1, and CCL2. Meanwhile, MAPK1, SOD1, TGFB1, and others can be only regulated by quercetin. It indicated that AM has the biological characteristics of multicomponent and multitarget in treating DN. Common target PPI network showed that the targets are also controlled by AM and DN, which revealed that AM could regulate the expression of DN-regulated targets and alleviate DN symptoms. VEGFA (degree = 41), TNF (degree = 40), IL-6 (degree = 39), MAPK1 (degree = 37), and CCL2 (degree = 35) might be the hub target of this network.

In order to predict the mechanism of AM in the treatment of DN, we analyzed the candidate targets by performing GO enrichment results, such as biological processes, molecular function, and cellular components. The top 20 GO terms (*P* < 0.05) were chosen from the 138 enrichment results, which indicated that the major hubs were significantly in multiple biological processes, including positive regulation of angiogenesis, lipopolysaccharide-mediated signaling pathway, angiogenesis, positive regulation of endothelial cell proliferation, and positive regulation of peptidyl-serine phosphorylation, as shown in Figure 4. Furthermore, molecular function enrichment analysis contains identical protein binding, cytokine activity, MAP kinase activity, heme binding, and peroxidase activity, as shown in Figure 5. The active targets involved are TNF, IL-6, MAPK1, PTGS2, and NOS3, which mainly concentrate on the molecular processes of immune regulation, oxidative stress, inflammatory response, etc. To some extent, this is consistent with the pathogenesis and mechanism of clinical DN. Meanwhile, cellular components consist of extracellular space, membrane raft, cytosol, extracellular matrix, caveola, Golgi apparatus, mitochondrion, and extracellular exosome, and many targets were ranked highly as potential related genes. This indirectly illustrated the complexity of the pathogenesis of DN and the damage to various cellular components. For instance, IL-6, CCL2, TNF, DPP4, and HIF-1*α* play a key regulatory role in this pathogenic process. Among them, DPP4 target inhibitors have been widely used in the treatment of diabetes mellitus, which has an important impact on reducing blood sugar in patients [32].

HIF-1 signaling pathway activates the expression of VEGFA in inflammatory response to counteract hypoxia and prevent kidney tissue damage in conjunction [33]. VEGFA contributes significantly in the pathogenesis of DN, and overexpression will promote the proliferation of microvascular endothelial cells and aggravate the occurrence of vascular disease. The research suggested that VEGFA could serve as biomarkers to identify the progression of DN and had higher expression in the plasma and urine of DN patients [34]. In addition, after treatment with medicine, not only FBG and Scr levels were decreased but also VEGF, HIF-1*α*, and TGF-*β*1 mRNA expression was downregulated in DN mice [35]. Meanwhile, NOS3, HIF-1*α*, and VEGFA play a crucial role in angiogenesis and endothelial cell proliferation. A further investigation showed that VEGF increased hTERT expression in a mechanism that implicates the PI3K/AKT/mTOR pathway and HIF-1 signaling pathway.

In the pathogenesis of DN, inflammatory cells will proliferate and differentiate, increase the infiltration of renal tissue cells, and secrete a large number of cytokines to mediate renal tissue damage. In addition, these inflammatory cells and cytokines secreted by them can stimulate and activate kidney cells, regulating the expression of chemokines, cytokines, adhesion molecules, and extracellular matrix components, and aggravate the pathological changes of kidney tissue. TNF-*α*, one of the main regulatory factors, which can induce the production of IL-1*β*, IL-6, and other cytokines, participates in the process of oxidative stress and inflammation and aggravation of renal tissue damage [36]. IL-6, as an autocrine growth factor of mesangial cells, can significantly stimulate mesangial proliferation. The level of IL-6 in urine can reflect the degree of glomerular and tubulointerstitial lesions, which has guiding significance for the diagnosis of diabetes mellitus [37]. However, treatment of diabetic rats might significantly inhibit the production and release of inflammatory cytokines IL-6, IL-1*β*, and TNF-*α* into the serum [38]. What is more, in agreement with the improved oxidative response, TNF-*α*, IL-1*β*, and ICAM-1 expression was all markedly blocked in DN mice [39]. Collectively, these inflammatory factors are mainly concentrated in rheumatoid arthritis, FoxO signaling pathway, toll-like receptor signaling pathway, and TNF signaling pathway, and aggravated the symptoms of DN to some extent.

MAPK1 is an important transmitter of signals from the cell surface to the nucleus in NOD-like receptor signaling pathway. It can activate the release of various inflammatory factors, chemokines, and adhesion factors mediated by NF-KB signaling pathways and aggravate the pathological changes of DN [40]. When inflammatory reaction happens, MAPK1 will promote IL-1, TNF-*α*, and IL-1*β* entry into glomerular cells by activating nod-like receptor signaling pathway and increase cell damage. CCL2 is a secretory protein that mediates the phosphorylation of C-terminal serine/threonine of receptor protein by binding to the N-terminal of CCR2 on target cell membrane [41]. It participates in various physiological and pathological activities of cells through different transmembrane signal transduction pathways. It can migrate chemokine cells and make immune cells migrate along the site where chemokine concentration increases. The release of chemokines can also stimulate the expression of inflammatory cytokines, such as IL-1, IL-6, and TNF-*α*, which can repair infection or tissue damage [42]. Therefore, these results also confirm that our screened targets are consistent with the literature reports, indicating that AM can play a therapeutic role in DN by regulating inflammatory response, oxidative stress, and other pathways.

## 5. Conclusion

In summary, AM has significant advantages in the treatment of DN, which is consistent with previous studies. At the same time, the biological functions of active ingredients and their corresponding targets of AM were analyzed by network pharmacological method, which further revealed the molecular biological mechanism of AM in treating DN. It has a significant value to provide theoretical basis for clinical treatment of DN.

## Figures and Tables

**Table 1:** Basic information of AM compound ingredients.

Mol ID	Molecule name	OB (%)	DL
MOL000211	Mairin	55.38	0.78
MOL000239	Jaranol	50.83	0.29
MOL000296	Hederagenin	36.91	0.75
MOL000033	(3S,8S,9S,10R,13R,14S,17R)-10,13-Dimethyl-17-[(2R,5S)-5-propan-2-yloctan-2-yl]-2,3,4,7,8,9,11,12,14,15,16,17-dodecahydro-1H-cyclopenta[a]phenanthren-3-ol	36.23	0.78
MOL000354	Isorhamnetin	49.6	0.31
MOL000371	3,9-Di-O-methylnissolin	53.74	0.48
MOL000379	9,10-Dimethoxypterocarpan-3-O-*β*-D-glucoside	36.74	0.92
MOL000380	(6aR,11aR)-9,10-Dimethoxy-6a,11a-dihydro-6H-benzofurano[3,2-c]chromen-3-ol	64.26	0.42
MOL000387	Bifendate	31.1	0.67
MOL000392	Formononetin	69.67	0.21
MOL000398	7-O-methylisomucronulatol	74.69	0.3
MOL000417	Calycosin	47.75	0.24
MOL000422	Kaempferol	41.88	0.24
MOL000433	FA	68.96	0.71
MOL000442	1,7-Dihydroxy-3,9-dimethoxy pterocarpene	39.05	0.48
MOL000098	Quercetin	46.43	0.28

**Table 2:** Information on potential targets and the topological attributes.

No.	Gene name	Protein name	UniProt ID	Degree
1	VEGFA	Vascular endothelial growth factor A	P15692	41
2	TNF	Tumor necrosis factor	P01375	40
3	IL-6	Interleukin-6	P05231	39
4	MAPK1	Mitogen-activated protein kinase 1	P28482	37
5	CCL2	C-C motif chemokine 2	P13500	35
6	PTGS2	Alcohol dehydrogenase 1B	P35354	34
7	IL-1*β*	Interleukin-1 beta	P01584	34
8	NOS3	Nitric oxide synthase, endothelial	P29474	34
9	JUN	Transcription factor AP-1	P05412	34
10	EGFR	Epidermal growth factor receptor	P00533	33
11	MAPK8	Mitogen-activated protein kinase 8	P45983	33
12	IL-10	Interleukin-10	P22301	31
13	PPARG	Peroxisome proliferator-activated receptor gamma	P37231	31
14	CXCL8	Interleukin-8	P10145	31
15	MMP2	72 kDa type IV collagenase	P08253	30
16	ESR1	Estrogen receptor	P03372	30
17	SERPINE1	Plasminogen activator inhibitor 1	P05121	30
18	TGFB1	Transforming growth factor beta-1	P01137	28
19	SIRT1	NAD-dependent deacetylase sirtuin-1	Q96EB6	28
20	MAPK14	Mitogen-activated protein kinase 14	Q16539	28
21	HIF-1*α*	Hypoxia-inducible factor 1-alpha	Q16665	28
22	CAV1	Caveolin-1	Q03135	26
23	SPP1	Osteopontin	P10451	25
24	MPO	Myeloperoxidase	P05164	25
25	MMP3	Stromelysin-1	P08254	24
26	KDR	Vascular endothelial growth factor receptor 2	P35968	24
27	NOS2	Nitric oxide synthase, inducible	P35228	23
28	SOD1	Superoxide dismutase (Cu-Zn)	P00441	23
29	NFE2L2	Nuclear factor erythroid 2-related factor 2	Q16236	21
30	GJA1	Gap junction alpha-1 protein	P17302	20
31	CDKN1A	Cyclin-dependent kinase inhibitor 1	P38936	19
32	IL-1A	Interleukin-1 alpha	P01583	16
33	PRKCA	Protein kinase C alpha type	P17252	15
34	PPARA	Peroxisome proliferator–activated receptor alpha	Q07869	14
35	IGF2	Insulin-like growth factor II	P01344	14
36	PTGS1	Prostaglandin G/H synthase 1	P23219	13
37	HSPA5	78 kDa glucose-regulated protein	P11021	12
38	DPP4	Dipeptidyl peptidase IV	P27487	11
39	PRKCB	Protein kinase C beta type	P05771	10
40	THBD	Thrombomodulin	P07204	10
41	AKR1B1	Aldose reductase	P15121	8
42	PON1	Serum paraoxonase/arylesterase 1	P27169	8
43	INSR	Insulin receptor	P06213	8
44	BCL2	Apoptosis regulator Bcl-2	P10415	7
45	E2F1	Transcription factor E2F1	Q01094	6
46	LYZ	Lysozyme	P61626	5
47	HTR2A	5-hydroxytryptamine 2A receptor	P28223	1
48	PRSS1	Trypsin-1	P07477	1

## Data Availability

The data used to support the findings of this study are available from the corresponding author upon request.

## Supplementary Materials

Supplementary 1 Table S1: Compound targets for each component in AM.

Supplementary 2 Table S2: Screening of common targets for DN and AM.
