# Supplementary material for: Uncovering the Mechanism of Astragalus membranaceus in the Treatment of Diabetic Nephropathy Based on Network Pharmacology
Source: J Diabetes Res. 2020 Mar 2;2020:5947304. doi: 10.1155/2020/5947304 (PMC7079250; doi:10.1155/2020/5947304)
Supplement: Supplementary 1 — Table S1: Compound targets for each component in AM. [file 5947304.f1.docx]

Table S1 Compound targets for each component in AM

| Compound | Gene |
| --- | --- |
| Mairin | PGR |
| [Jaranol](http://lsp.nwu.edu.cn/molecule.php?qn=239) | NOS2 |
| [Jaranol](http://lsp.nwu.edu.cn/molecule.php?qn=239) | PTGS1 |
| [Jaranol](http://lsp.nwu.edu.cn/molecule.php?qn=239) | AR |
| [Jaranol](http://lsp.nwu.edu.cn/molecule.php?qn=239) | SCN5A |
| [Jaranol](http://lsp.nwu.edu.cn/molecule.php?qn=239) | PTGS2 |
| [Jaranol](http://lsp.nwu.edu.cn/molecule.php?qn=239) | ESR2 |
| [Jaranol](http://lsp.nwu.edu.cn/molecule.php?qn=239) | DPP4 |
| [Jaranol](http://lsp.nwu.edu.cn/molecule.php?qn=239) | HSP90AA1 |
| [Jaranol](http://lsp.nwu.edu.cn/molecule.php?qn=239) | CDK2 |
| [Jaranol](http://lsp.nwu.edu.cn/molecule.php?qn=239) | CHEK1 |
| [Jaranol](http://lsp.nwu.edu.cn/molecule.php?qn=239) | PRSS1 |
| hederagenin | PGR |
| hederagenin | CHRM3 |
| hederagenin | CHRM1 |
| hederagenin | GABRA2 |
| hederagenin | GABRA3 |
| hederagenin | CHRM2 |
| hederagenin | ADRA1B |
| hederagenin | GABRA1 |
| hederagenin | GRIA2 |
| hederagenin | GABRA5 |
| hederagenin | RXRA |
| hederagenin | PDE3A |
| hederagenin | SLC6A2 |
| (3S,8S,9S,10R,13R,14S,17R)-10,13-dimethyl-17-[(2R,5S)-5-propan-2-yloctan-2-yl]-2,3,4,7,8,9,11,12,14,15,16,17-dodecahydro-1H-cyclopenta[a]phenanthren-3-ol | PGR |
| isorhamnetin | NOS2 |
| isorhamnetin | PTGS1 |
| isorhamnetin | AR |
| isorhamnetin | PTGS2 |
| isorhamnetin | ESR2 |
| isorhamnetin | DPP4 |
| isorhamnetin | HSP90AA1 |
| isorhamnetin | CDK2 |
| isorhamnetin | PRSS1 |
| isorhamnetin | GABRA1 |
| isorhamnetin | MAPK14 |
| isorhamnetin | GSK3B |
| isorhamnetin | PRKACA |
| isorhamnetin | PIM1 |
| isorhamnetin | CCNA2 |
| isorhamnetin | PYGM |
| isorhamnetin | PPARG |
| isorhamnetin | F7 |
| isorhamnetin | NOS3 |
| isorhamnetin | ACHE |
| isorhamnetin | MAOB |
| isorhamnetin | XDH |
| isorhamnetin | NCF1 |
| 3,9-di-O-methylnissolin | NOS2 |
| 3,9-di-O-methylnissolin | PTGS1 |
| 3,9-di-O-methylnissolin | PTGS2 |
| 3,9-di-O-methylnissolin | PRSS1 |
| 3,9-di-O-methylnissolin | CHRM3 |
| 3,9-di-O-methylnissolin | CHRM1 |
| 3,9-di-O-methylnissolin | ADRA1B |
| 3,9-di-O-methylnissolin | GABRA1 |
| 3,9-di-O-methylnissolin | RXRA |
| 3,9-di-O-methylnissolin | PDE3A |
| 3,9-di-O-methylnissolin | NOS3 |
| 3,9-di-O-methylnissolin | ACHE |
| 3,9-di-O-methylnissolin | ADRB1 |
| 3,9-di-O-methylnissolin | HTR3A |
| 3,9-di-O-methylnissolin | ADRA2C |
| 3,9-di-O-methylnissolin | ADRA1D |
| 3,9-di-O-methylnissolin | OPRM1 |
| 7-O-methylisomucronulatol | NOS2 |
| 7-O-methylisomucronulatol | PTGS1 |
| 7-O-methylisomucronulatol | AR |
| 7-O-methylisomucronulatol | SCN5A |
| 7-O-methylisomucronulatol | PTGS2 |
| 7-O-methylisomucronulatol | ESR2 |
| 7-O-methylisomucronulatol | PRSS1 |
| 7-O-methylisomucronulatol | CHRM3 |
| 7-O-methylisomucronulatol | CHRM1 |
| 7-O-methylisomucronulatol | CHRM2 |
| 7-O-methylisomucronulatol | ADRA1B |
| 7-O-methylisomucronulatol | RXRA |
| 7-O-methylisomucronulatol | PDE3A |
| 7-O-methylisomucronulatol | ESR1 |
| 7-O-methylisomucronulatol | PPARG |
| 7-O-methylisomucronulatol | PRKACA |
| 7-O-methylisomucronulatol | PIM1 |
| 7-O-methylisomucronulatol | CCNA2 |
| 7-O-methylisomucronulatol | NOS3 |
| 7-O-methylisomucronulatol | ADRB1 |
| 7-O-methylisomucronulatol | ADRA2C |
| 7-O-methylisomucronulatol | ADRB2 |
| 7-O-methylisomucronulatol | ADRA1D |
| 7-O-methylisomucronulatol | DRD1 |
| 7-O-methylisomucronulatol | KCNH2 |
| 7-O-methylisomucronulatol | F10 |
| 7-O-methylisomucronulatol | CHRM4 |
| 7-O-methylisomucronulatol | OPRD1 |
| 7-O-methylisomucronulatol | HTR2A |
| 7-O-methylisomucronulatol | ADRA1A |
| 7-O-methylisomucronulatol | SLC6A3 |
| 7-O-methylisomucronulatol | SLC6A4 |
| 9,10-dimethoxypterocarpan-3-O-β-D-glucoside | PTGS2 |
| (6aR,11aR)-9,10-dimethoxy-6a,11a-dihydro-6H-benzofurano[3,2-c]chromen-3-ol | NOS2 |
| (6aR,11aR)-9,10-dimethoxy-6a,11a-dihydro-6H-benzofurano[3,2-c]chromen-3-ol | PTGS1 |
| (6aR,11aR)-9,10-dimethoxy-6a,11a-dihydro-6H-benzofurano[3,2-c]chromen-3-ol | SCN5A |
| (6aR,11aR)-9,10-dimethoxy-6a,11a-dihydro-6H-benzofurano[3,2-c]chromen-3-ol | PTGS2 |
| (6aR,11aR)-9,10-dimethoxy-6a,11a-dihydro-6H-benzofurano[3,2-c]chromen-3-ol | HSP90AA1 |
| (6aR,11aR)-9,10-dimethoxy-6a,11a-dihydro-6H-benzofurano[3,2-c]chromen-3-ol | PRSS1 |
| (6aR,11aR)-9,10-dimethoxy-6a,11a-dihydro-6H-benzofurano[3,2-c]chromen-3-ol | CHRM1 |
| (6aR,11aR)-9,10-dimethoxy-6a,11a-dihydro-6H-benzofurano[3,2-c]chromen-3-ol | ADRA1B |
| (6aR,11aR)-9,10-dimethoxy-6a,11a-dihydro-6H-benzofurano[3,2-c]chromen-3-ol | GABRA1 |
| (6aR,11aR)-9,10-dimethoxy-6a,11a-dihydro-6H-benzofurano[3,2-c]chromen-3-ol | RXRA |
| (6aR,11aR)-9,10-dimethoxy-6a,11a-dihydro-6H-benzofurano[3,2-c]chromen-3-ol | ESR1 |
| (6aR,11aR)-9,10-dimethoxy-6a,11a-dihydro-6H-benzofurano[3,2-c]chromen-3-ol | ACHE |
| (6aR,11aR)-9,10-dimethoxy-6a,11a-dihydro-6H-benzofurano[3,2-c]chromen-3-ol | HTR3A |
| (6aR,11aR)-9,10-dimethoxy-6a,11a-dihydro-6H-benzofurano[3,2-c]chromen-3-ol | ADRB2 |
| (6aR,11aR)-9,10-dimethoxy-6a,11a-dihydro-6H-benzofurano[3,2-c]chromen-3-ol | ADRA1D |
| (6aR,11aR)-9,10-dimethoxy-6a,11a-dihydro-6H-benzofurano[3,2-c]chromen-3-ol | CHRM4 |
| (6aR,11aR)-9,10-dimethoxy-6a,11a-dihydro-6H-benzofurano[3,2-c]chromen-3-ol | CHRNA7 |
| Bifendate | PTGS1 |
| Bifendate | PTGS2 |
| Bifendate | HSP90AA1 |
| formononetin | NOS2 |
| formononetin | PTGS1 |
| formononetin | AR |
| formononetin | PTGS2 |
| formononetin | ESR2 |
| formononetin | DPP4 |
| formononetin | HSP90AA1 |
| formononetin | CDK2 |
| formononetin | CHEK1 |
| formononetin | PRSS1 |
| formononetin | CHRM1 |
| formononetin | RXRA |
| formononetin | PDE3A |
| formononetin | ESR1 |
| formononetin | PPARG |
| formononetin | MAPK14 |
| formononetin | GSK3B |
| formononetin | PRKACA |
| formononetin | PIM1 |
| formononetin | CCNA2 |
| formononetin | NOS3 |
| formononetin | ACHE |
| formononetin | MAOB |
| formononetin | ADRB2 |
| formononetin | ADRA1A |
| formononetin | SLC6A3 |
| formononetin | SLC6A4 |
| formononetin | blaZ |
| formononetin | JUN |
| formononetin | PPARG |
| formononetin | IL4 |
| formononetin | SIRT1 |
| Calycosin | NOS2 |
| Calycosin | PTGS1 |
| Calycosin | AR |
| Calycosin | PTGS2 |
| Calycosin | ESR2 |
| Calycosin | DPP4 |
| Calycosin | HSP90AA1 |
| Calycosin | CDK2 |
| Calycosin | CHEK1 |
| Calycosin | PRSS1 |
| Calycosin | RXRA |
| Calycosin | PDE3A |
| Calycosin | ESR1 |
| Calycosin | PPARG |
| Calycosin | MAPK14 |
| Calycosin | GSK3B |
| Calycosin | PRKACA |
| Calycosin | PIM1 |
| Calycosin | CCNA2 |
| Calycosin | ADRB2 |
| kaempferol | NOS2 |
| kaempferol | PTGS1 |
| kaempferol | AR |
| kaempferol | PTGS2 |
| kaempferol | ESR2 |
| kaempferol | DPP4 |
| kaempferol | HSP90AA1 |
| kaempferol | CDK2 |
| kaempferol | CHEK1 |
| kaempferol | PRSS1 |
| kaempferol | RXRA |
| kaempferol | PDE3A |
| kaempferol | ESR1 |
| kaempferol | PPARG |
| kaempferol | MAPK14 |
| kaempferol | GSK3B |
| kaempferol | PRKACA |
| kaempferol | PIM1 |
| kaempferol | CCNA2 |
| kaempferol | ADRB2 |
| FA | CDK2 |
| FA | GSK3B |
| 1,7-Dihydroxy-3,9-dimethoxy pterocarpene | PTGS2 |
| 1,7-Dihydroxy-3,9-dimethoxy pterocarpene | HSP90AA1 |
| 1,7-Dihydroxy-3,9-dimethoxy pterocarpene | PRSS1 |
| 1,7-Dihydroxy-3,9-dimethoxy pterocarpene | RXRA |
| quercetin | PTGS1 |
| quercetin | AR |
| quercetin | SCN5A |
| quercetin | PTGS2 |
| quercetin | DPP4 |
| quercetin | HSP90AA1 |
| quercetin | PRSS1 |
| quercetin | GABRA1 |
| quercetin | RXRA |
| quercetin | PPARG |
| quercetin | PRKACA |
| quercetin | F7 |
| quercetin | NOS3 |
| quercetin | ACHE |
| quercetin | MAOB |
| quercetin | XDH |
| quercetin | NCF1 |
| quercetin | ADRB2 |
| quercetin | KCNH2 |
| quercetin | F10 |
| quercetin | JUN |
| quercetin | PPARG |
| quercetin | BCL2 |
| quercetin | TNF |
| quercetin | MMP1 |
| quercetin | CDK1 |
| quercetin | HMOX1 |
| quercetin | CYP3A4 |
| quercetin | ICAM-1 |
| quercetin | SELE |
| quercetin | VCAM1 |
| quercetin | CYP1B1 |
| quercetin | ALOX5 |
| quercetin | GSTP1 |
| quercetin | AHR |
| quercetin | INSR |
| quercetin | DIO1 |
| quercetin | AKR1B1 |
| quercetin | MMP3 |
| quercetin | EGFR |
| quercetin | VEGFA |
| quercetin | CCND1 |
| quercetin | FOS |
| quercetin | CDKN1A |
| quercetin | CASP9 |
| quercetin | PLAU |
| quercetin | MMP2 |
| quercetin | MMP-9 |
| quercetin | MAPK1 |
| quercetin | IL10 |
| quercetin | IL6 |
| quercetin | TP53 |
| quercetin | ODC1 |
| quercetin | CASP8 |
| quercetin | SOD1 |
| quercetin | PRKCA |
| quercetin | HIF1A |
| quercetin | HSPA5 |
| quercetin | ERBB2 |
| quercetin | ACC1 |
| quercetin | CAV1 |
| quercetin | MYC |
| quercetin | F3 |
| quercetin | GJA1 |
| quercetin | IL1B |
| quercetin | CCL2 |
| quercetin | PTGER3 |
| quercetin | CXCL8 |
| quercetin | PRKCB |
| quercetin | BIRC5 |
| quercetin | TGFB1 |
| quercetin | MGAM |
| quercetin | IL2 |
| quercetin | CCNB1 |
| quercetin | PLAT |
| quercetin | THBD |
| quercetin | SERPINE1 |
| quercetin | IFNG |
| quercetin | IL1A |
| quercetin | MPO |
| quercetin | NFE2L2 |
| quercetin | CHEK2 |
| quercetin | CLDN4 |
| quercetin | PPARA |
| quercetin | CHUK |
| quercetin | SPP1 |
| quercetin | RUNX2 |
| quercetin | E2F1 |
| quercetin | E2F2 |
| quercetin | ACPP |
| quercetin | CTSD |
| quercetin | TMEM219 |
| quercetin | IGF2 |
| quercetin | CD40LG |
| quercetin | IRF1 |
| quercetin | ERBB3 |
| quercetin | PON1 |
| quercetin | NPEPPS |
| quercetin | NKX3-1 |
